# A modified Inflammatory Bowel Disease questionnaire and the Vaizey Incontinence questionnaire are simple ways to identify patients with significant gastrointestinal symptoms after pelvic radiotherapy

**DOI:** 10.1038/sj.bjc.6602552

**Published:** 2005-04-26

**Authors:** F A Olopade, A Norman, P Blake, D P Dearnaley, K J Harrington, V Khoo, D Tait, C Hackett, H J N Andreyev

**Affiliations:** 1 Department of Medicine and Therapeutics, Imperial College Faculty of Medicine, Chelsea and Westminster Hospital, 369 Fulham Road, London SW10 9NH, UK; 2 Department of Computing, The Royal Marsden Hospital, London and Sutton, UK; 3 Department of Radiotherapy, The Royal Marsden Hospital, London and Sutton, UK

**Keywords:** pelvic radiotheraphy, IBDQ, Vaizey, toxicity, LENT SOMA

## Abstract

After radiotherapy for pelvic cancer, chronic gastrointestinal problems may affect quality of life (QOL) in 6–78% of patients. This variation may be due to true differences in outcome in different diseases, and may also represent the inadequacy of the scales used to measure radiotherapy-induced gastrointestinal side effects. The aim of this study was to assess whether outcome measures used for nonmalignant gastrointestinal disease are useful to detect gastrointestinal morbidity after radiotherapy. Results obtained from a Vaizey Incontinence questionnaire and a modified Inflammatory Bowel Disease questionnaire (IBDQ) – both patient completed – were compared to those from a staff administered Late Effects on Normal Tissue (LENT) – Subjective, Objective, Management and Analytic (SOMA) questionnaire in patients who had completed radiotherapy for a pelvic tumour at least 3 months previously. In all, 142 consecutive patients were recruited, 72 male and 70 female, median age 66 years (range 26–90 years), a median of 27 (range 3–258) months after radiotherapy. In total, 62 had been treated for a gynaecological, 58, a urological and 22, a gastrointestinal tract tumour. Of these, 21 had undergone previous gastrointestinal surgery and seven suffered chronic gastrointestinal disorders preceding their diagnosis of cancer. The Vaizey questionnaire suggested that 27% patients were incontinent for solid stools, 35% for liquid stools and 37% could not defer defaecation for 15 min. The IBDQ suggested that 89% had developed a chronic change in bowel habit and this change significantly affected 49% patients: 44% had more frequent or looser bowel movements, 30% were troubled by abdominal pain, 30% were troubled by bloating, 28% complained of tenesmus, 27% were troubled by their accidental soiling and 20% had rectal bleeding. At least 34% suffered emotional distress and 22% impairment of social function because of their bowels. The small intestine/colon SOMA median score was 0.1538 (range 0–1) and the rectal SOMA median score was 0.1428 (range 0–1). Pearson's correlations for the IBDQ score and small intestine/colon SOMA score was −0.630 (*P*<0.001), IBDQ and rectum SOMA −0.616 (*P*<0.001), IBDQ and Vaizey scores −0.599 (*P*<0.001), Vaizey and small intestine/colon SOMA 0.452 (*P*<0.001) and Vaizey and rectum SOMA 0.760 (*P*<0.001). After radiotherapy for a tumour in the pelvis, half of all patients develop gastrointestinal morbidity, which affects their QOL. A modified IBDQ and Vaizey questionnaire are reliable in assessing new gastrointestinal symptoms as well as overall QOL and are much easier to use than LENT SOMA.

Quality of life (QOL) measures are important in the management of patients with malignant disease. These measures must be reliable and convenient to use and should assess physical, functional, social and emotional well-being (Cella *et al*, 2002).

Cancer treatments are becoming increasingly successful at obtaining long-term palliation and cure. In order to define and offer optimal treatments and perhaps to define which patients require further specialist evaluation or support after treatment, it is very important to measure QOL and chronic adverse treatment effects accurately.

Radiotherapy is used in the treatment of four out of 10 cancer patients. In the UK, this includes about 12 000 patients who receive radiotherapy for pelvic cancer, mainly with curative intent. However, the frequency with which pelvic radiotherapy leads to side effects affecting QOL is not known. The literature suggests that it ranges from 6 to 78% of patients (Kollmorgen *et al*, 1994; Potosky *et al*, 2000; Gami *et al*, 2003; Kozelsky *et al*, 2003). This variation may reflect a true difference in chronic toxicity in different diseases using different techniques. It seems more likely, however, that this degree of variation reflects the inadequacy of currently used toxicity scales. Indeed, the CTC Version 2, the Dische system, Franco-Italian Glossary, UCLA Cancer Index and RTOG/EORTC have all been shown to have shortcomings (Shakespeare *et al*, 1998; Yeoh *et al*, 2000; Anacak *et al*, 2001; Livsey *et al*, 2002; Trotti, 2002). More recently, the Late Effects Normal Tissue (LENT) SOMA questionnaire (Pavy *et al*, 1995; Rubin *et al*, 1995) has been proposed, and while overcoming many of the problems of earlier questionnaires, it is much more complex and requires a lengthy, structured interview with a patient, which is often not feasible.

In general, evaluations of gastrointestinal toxicity after radiotherapy have failed to draw on experience gained by gastroenterologists in designing scoring systems to judge severity of nonmalignant gastrointestinal diseases. Inflammatory diseases such as ulcerative colitis and Crohn's disease can produce many of the same symptoms as radiotherapy toxicity. Of the many questionnaires developed for evaluation of outcome in these conditions, the Inflammatory Bowel Disease questionnaire (IBDQ) does not require an interview and can be completed by a patient within a few minutes. It is a good predictor of severity of disease and in addition provides a score for QOL (Guyatt *et al*, 1989). The questions used in the IBDQ reflect those aspects of the disease that are recognised by patients and health professionals alike as frequent and important problems. These include bowel symptoms, systemic symptoms, emotional function and social function. In addition, it is clear that patients find the IBDQ easy to understand. A second questionnaire, the Vaizey incontinence score, again completed by most patients within 2–3 min has been shown to outperform other questionnaires as a simple and accurate way of detecting faecal incontinence (Vaizey *et al*, 1999).

The aim of this study was to assess whether the IBDQ and Vaizey Incontinence questionnaire, two simple patient completed questionnaires, could accurately assess the degree of gastrointestinal chronic toxicity and disability experienced after pelvic radiotherapy.

## PATIENTS AND METHODS

The population studied had completed radiotherapy for a pelvic cancer 3 months or more previously. Patients who had received radiotherapy for gynaecological, bladder, prostate or rectosigmoid or anal cancers were recruited from outpatient follow-up clinics at the Royal Marsden Hospital in London and Surrey, UK. Eligible patients were enrolled after providing informed consent. The study was reviewed and approved by the Royal Marsden Research and Ethics Committees.

Patients' age, primary tumour site, date of diagnosis, current disease status and any previous bowel surgery or chronic gastrointestinal disease were recorded. Each patient was asked to fill in the IBDQ and Vaizey questionnaires. Then an assessment of bowel toxicity was made using the LENT SOMA questionnaire in a structured interview.

The IBDQ contains 32 questions. Each question is scored 1–7 according to the severity of symptoms. Patients score 7 if the symptom asked about is absent or has not changed since before radiotherapy. They score 1 if that symptom is the worst possible. Thus, the highest attainable score is 224, suggesting that the patient is asymptomatic and the lowest attainable score is 32, suggesting that the patient is very severely symptomatic. The IBDQ as initially described includes five questions on systemic symptoms joint, skin or eye problems – which some patients with inflammatory bowel disease sometimes develop. These questions were modified before use in this study. The modified IBDQ is shown in Table 1. In addition to an overall symptom score, the IBDQ provides a QOL score by assessing emotional and social function.

The Vaizey Incontinence questionnaire consists of seven questions. These are scored as shown in Table 2. A score of 0 suggests no problems with bowel continence, and a score of 24 suggests very severe problems with incontinence.

The LENT SOMA scores are obtained after completing a series of questions, which grade subjective and objective symptoms and score for the requirement for medical intervention. Small bowel/colon and rectal scores can then be summed and divided by the total number of questions to give a overall score of between 0 (no symptoms) and 5 (fatal toxicity).

### Statistics

We hypothesised that as patients with different types of pelvic cancer receive different types of therapy, they might develop a different profile of symptoms following therapy. To examine whether these questionnaires measured chronic toxicity accurately for different pelvic cancers, we calculated that 47 gynaecological or bladder patients, 47 prostate patients and 47 lower gastrointestinal patients would be sufficient to allow a correlation coefficient of *r*=0.45 to be detected within each group with 90% power. If the groups were then combined, this would allow a correlation of *r*=0.25 to be detected. Associations between categorical data were examined using the *χ*^2^ test.

## RESULTS

### Patient characteristics

During July and August 2003, 142 consecutive patients were recruited onto the study; 62 had been treated for a gynaecological cancer, 58 for a urological cancer and 22 for a cancer in the gastrointestinal tract within the pelvis. Their characteristics are shown in Table 3. Time from diagnosis was between 5 and 258 months (median 32 months). All 142 patients had started radiotherapy at least 3 months beforehand (median 27 months, range 3–258 months). Each interview took approximately 15 min.

### Radiotherapy technique and dose prescription

A variety of radiotherapy techniques were used in different patient groups according to tumour type. The typical protocols are summarised in Table 4, although treatments and doses were individualised for each patient. External beam radiotherapy was delivered by a linear accelerator delivering megavoltage photons at an energy of 4–10 MV.

### The Vaizey Incontinence questionnaire

The results obtained from the Vaizey questionnaire are shown in Table 5. These suggest that overall 55 (39%) patients were incontinent for solid or liquid stools, and that 23% (55% of these) felt their incontinence altered their lifestyle. Faecal incontinence occurred in 50% of those treated for gynaecological cancer (95% confidence intervals 37–63%) and in 50% of those with a gastrointestinal cancer (95% confidence intervals 28.2–71.8%), and this was significantly more common (gynaecological *vs* urology, *P*=0.003 and GI *vs* urology, *P*=0.026) than in the 24% (95% confidence intervals 13.9–37.2%) patients who had been treated for a urological cancer.

The median score on the Vaizey questionnaire was 3.5 (range 0–24). In all, 46 patients had no faecal continence problems, if they are excluded, and the other 96 patients had a median score of 5 (range 1–24).

### The modified IBDQ

**Figure 1:**
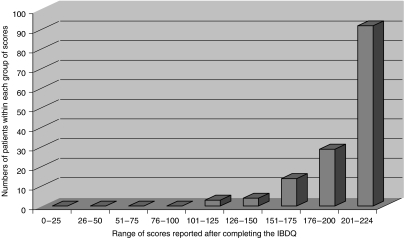
Numbers of patients within each range of scores using the IBDQ.

The results and range obtained from the IBDQ are shown in Tables 6a–d and Figure 1. The data suggest that 127 patients (89%) had noticed a change of varying severity in their bowel habit. The urology patients reported fewer symptoms than the gynaecology and gastrointestinal patients.

The median score on the IBDQ for all the patients was 212 (range 101–224). Among the symptomatic patients, the median score was 207 (range 101–223).

### LENT SOMA questionnaire (Table 7)

The median score for all patients on the small intestine/colon SOMA was 0.1538 (range 0–1) and the rectal SOMA median score was 0.1428 (range 0–1). For those patients who were symptomatic, the median score on the small intestine/colon SOMA was 0.2307 (range 0.0769–1) and the rectal SOMA was 0.2143 (range 0.0714–1).

### Correlations between IBDQ and Vaizey questionnaire with LENT SOMA (Table 8)

The small intestine/colon SOMA median score was 0.1538 (range 0–1) and the rectal SOMA median score was 0.1428 (range 0–1). Pearson's correlations for the IBDQ score and small intestine/colon SOMA score was −0.630 (*P*<0.001), IBDQ and rectum SOMA −0.616 (*P*<0.001), IBDQ and Vaizey scores −0.599 (*P*<0.001), Vaizey and small intestine/colon SOMA 0.452 (*P*<0.001) and Vaizey and rectum SOMA 0.760 (*P*<0.001).

### Scores for patients with previous bowel surgery or chronic gastrointestinal disorders

Of the 142 patients, 28 had had either a previous bowel disorder or bowel surgery before commencement of radiotherapy. These patients tended to have increased gastrointestinal morbidity as defined by two questionnaires. Compared to those without a previous gastrointestinal history, they ad a median score of 5 instead of 1 (Vaizey) and a score of 0.21 *vs* 0.071 (small bowel/colon SOMA) and 0.15 *vs* 0.77 (Rectal SOMA). However, they had an improved IBDQ score (213 *vs* 203).

## DISCUSSION

This study shows that there is a highly significant correlation between the degree of gastrointestinal dysfunction recorded in patients who had completed pelvic radiotherapy at least 3 months previously when two simple, patient self-administered questionnaires validated for benign diseases were used in comparison to scores recorded when the staff administered LENT SOMA questionnaire was used. Furthermore, these data suggest that at least half of all patients who have received radiotherapy for a pelvic tumour still suffer from bowel problems affecting their QOL 3 or more months after radiotherapy. The data suggest that symptoms may be more common after therapeutic irradiation for gynaecological and gastrointestinal tumours than for urological tumours.

The optimal combination of surgery, chemotherapy and radiotherapy for pelvic cancer is frequently controversial. In the past, treatment choices have usually been recommended on the basis of the likelihood of cure. And while cure clearly remains the central issue, if there is a high likelihood of long-term survival and varying regimens have equal likelihood of success, it becomes imperative that long-term toxicity and effect of the treatment on QOL is also considered. Robust data on the long-term gastrointestinal toxicity of radiotherapy used to treat pelvic cancers are scanty. This is mainly because the methods used to assess gastrointestinal toxicity historically have been inadequate. More recent questionnaires have proved too complex for clinical practice. The few prospective studies that have used careful assessment have suggested that long-term bowel toxicity is of the order we have documented here (Lundby *et al*, 1997; Dahlberg *et al*, 1998; Kozelsky *et al*, 2003), but the identification of patients with significant gastrointestinal toxicity in clinical practice frequently remains inadequate.

These questionnaires identified bowel dysfunction in many patients. The significance of this dysfunction is more difficult to determine. Physicians' perceptions of what constitutes severe toxicity and its effect on QOL may differ from patients. The clinical implications of an abnormal Vaizey score, however small, may be significant for an individual patient. The whole cohort reported a median Vaizey score of 3.5 and when the patients with no incontinence were excluded, the median score was still only 5 (range 1–24). While this may seem modest, only two points can mean the difference between a patient complaining of incontinence to liquid stool ‘daily’ or ‘sometimes’. From a patient's perspective, this can mean the ability to do one's own weekly shopping, or relying on help from others, which may take on social implications. It is not clear why when 35% of patients are incontinent for stools, only 23% feel that this incontinence affects their lifestyle. On the other hand, faecal incontinence scores by themselves may not be helpful. If a patient has severe urgency, but never leaves the house, they may be rarely incontinent, but their QOL may be poor. For example, 11% of our patients had not gone somewhere because there was no lavatory nearby and 28% had been worried about finding a lavatory when out.

The clinical implication of a single abnormal IBDQ score is also unclear. However, there are excellent data from patients with inflammatory bowel disease showing that if their disease is quiescent, that they will consistently score their QOL within a very narrow range of scores. Thus, if sequential scores deteriorate with time, this is a reliable indicator of disease activity producing gastrointestinal, systemic and psychological symptoms. This has been demonstrated both in a primary-care setting as well as in hospital practice (Guyatt *et al*, 1989; Yoshida, 1999; Cheung *et al*, 2000; Rubin *et al*, 2004). In other words, in the setting of benign inflammatory disease, this questionnaire is highly reliable, and while we have not tested the reproducibility of scores in patients with stable symptoms in this study, there is little reason to think that this group of patients should score change in symptoms differently to those with inflammatory bowel disease. However, this study suggests that in the symptomatic patients, the median IBDQ score was 207 (range 101–223). Further research is required to determine whether this means that symptoms were indeed relatively minor or, as has been demonstrated before, that patients minimise and accept their symptoms when they believe they are the inevitable consequences of radiotherapy treatment, of being old or that there is nothing that can be done (Faithfull, 1995).

Perhaps for the oncologist in clinical practice, the most useful way therefore, to use the IBDQ and Vaizey questionnaires in patients undergoing radiotherapy would be to record a score before treatment starts and then record sequential scores, which if deteriorating are likely to reflect significant gastrointestinal morbidity. For patients in clinical trials, this study suggests that the IBDQ may offer a significantly more sensitive indicator of gastrointestinal disease and its effect on QOL than RTOG or LENT SOMA.

LENT SOMA system tackles both objective and subjective morbidity, and unlike IBDQ, attempts to integrate it with data from clinical tests and medical interventions. However, it then restricts its conclusions to one of five bands, which blunts its sensitivity in describing subtle but potentially important problems. In addition, it is difficult to use with 11% of patients failing to complete their LENT SOMA questionnaire in one prospective study (Routledge *et al*, 2003).

This study failed to recruit its target number of gastrointestinal tumour patients because unusually low numbers of these patients attended clinic over the 8 weeks during which this study was carried out. Therefore in this group, who may have additional gastrointestinal symptoms from their tumour or previous surgery, it is particularly difficult to be sure that the IBDQ is a sensitive measure of radiation toxicity. The reduced toxicity we noted in the prostate patients may reflect that treatment for prostate cancer leads to less volume of bowel exposed to radiotherapy. If this is the case, it is interesting to speculate whether this advantage will be maintained if IMRT becomes widely used. However, there is some evidence that men may be more reluctant than women to report gastrointestinal symptoms generally, and when they do report them, it is generally at a lower level. Therefore, it is important not to assume toxicity is negligible just because patients are slow to report problems.

In summary, this study suggests that simple, long established questionnaires used in benign gastroenterological diseases and validated in primary-care and hospital settings and across cultural divides appear to be applicable to oncological patients. Their simplicity and precision makes them ideal for clinical practice to identify patients who require specialist help or in the clinical trial setting, to provide a sensitive measure of gastrointestinal toxicity.

## Figures and Tables

**Table 1:** The modified IBDQ

	**In the last 2 weeks please tell us how often you have:**	**More than ever before**	**Extremely frequently**	**Very frequently**	**Moderate increase in frequency**	**Some increase in frequency**	**Slight increase in frequency**	**Not at all/normal**
1	Had your bowel open?							
2	Felt tired and worn out?							
3	Felt frustrated, impatient or restless?							
4	Been unable to do what you want because of your bowels?							
5	Had loose bowel movements?							
6	Worried about your energy levels?							
7	Worried about having to have something done about your bowels?							
8	You had to cancel an engagement because of your bowels?							
9	Been troubled by pain in your bottom?							
10	Felt generally unwell?							
11	Worried about not being able to find a lavatory?							
12	Been prevented doing leisure or sports by your bowels?							
13	Been troubled by pain in your tummy or bottom?							
14	Been waking at night or having difficulty sleeping?							
15	Been depressed or discouraged?							
16	Not gone somewhere because there is no lavatory nearby?							
17	Passed large amounts of gas?							
18	Worried about getting to the weight you would like?							
19	Worried about your illness?							
20	Been troubled by bloating?							
21	Been relaxed and free of tension?							
22	Had a problem with bleeding from your bottom?							
23	Been embarrassed about your bowels?							
24	Felt like you need to have your bowels open but nothing happens?							
25	Felt tearful and upset?							
26	Been troubled by accidental soiling?							
27	Felt angry as a result of your bowel problems?							
28	Felt limited in sexual activity because of your bowels?							
29	Felt disgusted about your bowel problems?							
30	Felt irritable?							
31	Experienced lack of understanding from others?							
32	Felt satisfied, happy or pleased with your life?							

**Table 2:** Vaizey questionnaire

	**Never**	**Rarely**	**Sometimes**	**Weekly**	**Daily**
Incontinence for solid stool	0	1	2	3	4
Incontinence for liquid stool	0	1	2	3	4
Incontinence for gas	0	1	2	3	4
Alteration to lifestyle	0	1	2	3	4
				
		No		Yes	
Need to wear a pad or plug		0		2	
Taking constipating medicines		0		2	
Lack of ability to defer defecation for 15 min		0		4	

Never=no episodes in past 4 weeks;

Rarely=1 episode in past 4 weeks;

Sometimes=>1 episode in past 4 weeks but <1 a week;

Weekly=1 or more episodes a week but <1 a day;

Daily=1 or more episodes a day;

Minimum score=0=perfect continence;

Maximum score=24=total incontinence.

**Table 3:** Patient characteristics

	**Gynaecology**	**Urology**	**GI**	**Total**
*No. of patients (%)*	62 (44)	58 (41)	22 (15)	142
Median age (years)	59.5	69	67	66
Range	27–90	26–83	51–80	26–90
				
*Tumour site*				
Anal	—	—	8	
Caecum	—	—	3	
Colon	—	—	1	
Rectum	—	—	9	
Other	—	—	1	
Cervix	22	—	—	
Uterus	32	—	—	
Vulva	5	—	—	
Vagina	2	—	—	
Bartholin's	1	—	—	
Prostate	—	56	—	
Seminoma	—	2	—	
				
*Median time since diagnosis (months)*	31	48	36	32
Range	5–258	8–133	7–159	5–258
*Median time since radiotherapy* *(months)*	26	32	23	27
Range	4–258	3–119	3–157	3–258
				
*Bowel history*				
Anal stretch	—	1	—	1
Colitis	1	—	—	1
Diverticulitis	—	2	1	3
Haemorrhoidectomy	—	3	—	3
Irritable bowel	—	1	1	2
Intussusception	—	1	—	1
Polypectomy	—	1	—	1
Bowel resection	2	2	12	16
None	59	47	8	114
				
*Current disease status*				
No evidence	54	48	19	121
Quiescent	4	8	2	14
Progressive	4	2	1	7

GI=gastrointestinal.

**Table 4:** Typical radiotherapy parameters for pelvic treatment sites

**Tumour site**	**Patient position**	**Planning technique**	**Typical beam arrangement**	**Total dose (Gy)**	**Fraction size (Gy)**	**Approx field**	**Brachytherapy**
Bladder	Supine	Conformal CT	Anterior	64	2	10 × 10	—
			Opposed laterals				
			AP : PA : Lats				
							
Prostate	Supine	Conformal CT	3 field:	64–70	2	9 × 9	—
		2 phase	Anterior				
			Opposed laterals			6 × 6	
			6 field:	±boost to 74			
			Laterals				
			Ant/post obliques				
							
Testis	Supine	Simulated	AP:PA	20–30	2	10 × 15–30	
		Bony anatomy	Para-aortic dogleg				
							
Anal canal	Prone	Simulated	AP:PA	45	1.8	15 × 20	—
		Bony anatomy	3 field	15			
			Direct electron implant				
							
Rectum	Prone	Conformal CT	3 field:	45	1.8	17 × 17	—
		2 phase	Post			17 × 10	
			Opposed laterals	5.4–9	1.8	10 × 10	
							
Uterus	Supine	Simulated	3 field:	45	1.8	16 × 16	Ir-192
		Bony anatomy	Anterior				HDR
			Opposed laterals				8 Gy in two insertions at 0.5 cm
							
Vulva/vagina	Supine	Simulated	AP:PA	45	1.8	15 × 20	—
		Bony anatomy	3 field				
							
Cervix	Supine	Simulated	3 field:	45–50.4	1.8	24 × 16	Cs 137
		Bony anatomy	Anterior opposed laterals				LDR 22.5–27 Gy to point A

HDR=high-dose rate; LDR=low-dose rate; AP=antero-posterior; PA=postero-anterior; CT=computer tomography; Cs=caesium.

**Table 5:** Vaizey questionnaires (proportion of patients with positive results)

	**Gynaecology (% of all gynaecology patients)**	**Urology (% of all urology patients)**	**GI (% of all gastrointestinal patients)**	**Total (% of all patients)**
Incontinent for solid stools	19 (31)	9 (15)	10 (45)	38 (22)
Incontinent for liquid stools	29 (47)	11 (19)	10 (45)	50 (35)
Incontinence for gas	36 (58)	22 (38)	12 (55)	70 (49)
Incontinence affecting lifestyle	19 (31)	7 (12)	6 (27)	32 (22)
				
Need to wear a pad or plug	10 (16)	2 (3)	4 (18)	16 (11)
Need for constipating medicines	10 (16)	5 (9)	6 (27)	21 (15)
Cannot defer defecation for 15 min	33 (53)	12 (21)	8 (36)	89 (63)

GI=gastrointestinal.

**Table 6:** IBDQ (a) proportion of patients with bowel symptoms, (b) proportion of patients with systemic symptoms, (c) proportion of patients with emotional symptoms and (d) proportion of patients with social symptoms

	**Gynaecology (% of all gynaecology patients)**	**Urology (% of all urology patients)**	**GI (% of all gastrointestinal patients)**	**Total (% of all patients)**
*(a) IBDQ (proportion of patients with bowel symptoms)*				
Increase in bowel frequency	35 (56)	14 (24)	13 (59)	62 (44)
Loose motions	32 (52)	19 (32)	11 (50)	62 (44)
Pain in abdomen or bottom	30 (48)	9 (16)	4 (18)	43 (30)
Passing large amount of gas	31 (50)	23 (40)	12 (55)	66 (46)
Feeling of bloating	28 (45)	9 (16)	7 (32)	44 (31)
Rectal bleeding	14 (23)	9 (16)	5 (23)	28 (20)
Tenesmus	19 (31)	13 (22)	8 (36)	40 (28)
Accidental soiling	23 (37)	7 (2)	9 (41)	39 (27)
Feeling disgusted about bowel problems	12 (19)	1 (2)	3 (14)	16 (11)
				
*(b) IBDQ (proportion of patients with systemic symptoms)*				
Felt tired and worn out	38 (61)	27 (47)	9 (41)	74 (52)
Worried about energy levels	35 (55)	19 (33)	6 (27)	59 (42)
Felt generally unwell	22 (35)	8 (14)	3 (14)	33 (23)
Waking at night or having difficulty sleeping	27 (44)	17 (29)	12 (55)	56 (39)
Worried about getting to the weight you would like	21 (34)	17 (29)	5 (23)	43 (30)
				
*(c) IBDQ (proportion of patients with emotional symptoms)*				
Felt frustrated, impatient or restless	25 (40)	16 (27)	7 (32)	48 (34)
Worried about having something done about your bowels	12 (8)	3 (2)	6 (27)	21 (15)
Worried about not being able to find a lavatory	13 (21)	9 (15)	8 (36	30 (21)
Been depressed or discouraged	20 (32)	14 (24)	8 (36)	42 (30)
Worried about your illness	23 (16)	12 (21)	7 (32)	42 (30)
Relaxed and free of tension	11 (18)	5 (9)	5 (23)	21 (15)
Been embarrassed about your bowels	18 (30)	6 (10)	3 (14)	27 (19)
Felt tearful or upset	18 (30)	8 (14)	8 (36)	34 (24)
Felt angry as a result of bowel problems	12 (19)	1 (2)	2 (9)	15 (11)
Felt irritable	30 (48)	12 (21)	6 (27)	48 (34)
Experienced lack of understanding from others	7 (11)	3 (5)	3 (14)	13 (9)
Not felt satisfied, happy or pleased with your life	14 (23)	3 (5)	1 (5)	18 (29)
				
*(d) IBDQ (proportion of patients with social symptoms)*				
Unable to do what you want because of your bowels	22 (35)	5 (9)	4 (18)	31 (22)
Cancelled an engagement because of your bowels	7 (11)	1 (2)	2 (9)	10 (7)
Not done leisure or sport because of your bowels	14 (23)	2 (3)	3 (14)	19 (13)
Not gone somewhere because there is no lavatory nearby	12 (19)	2 (3)	2 (9)	16 (11)
Limited in sexual activity because of your bowels	7 (11)	0 (0)	1 (5)	8 (6)

IBDQ=Inflammatory Bowel Disease questionnaire; GI=gastroinstestinal.

**Table 7:** Scores of (a) the entire cohort using Vaizey, IBDQ and LENT SOMA and (b) only the symptomatic patients using Vaizey, IBDQ and LENT SOMA

	**Gynaecology**	**Urology**	**GI**	**Total**
*(a) Scores of the entire cohort using Vaizey, IBDQ and LENT SOMA*				
Vaizey				
Median score	5	1	4	3.5
Range	0–24	0–16	0–22	0–24
				
IBDQ				
Median score	196.5	218.5	214	212
Range	101–224	158–224	156–224	101–224
				
Small bowel SOMA				
Median score	0.2307	0.7692	0.1923	0.1538
Range	0–1	0–0.4615	0–0.5385	0–1
				
Rectal SOMA				
Median score	0.1786	0.0714	0.2143	0.1428
Range	0–1	0–0.7143	0–0.9286	0–1
				
*(b) Scores of only the symptomatic patients using Vaizey, IBDQ and LENT SOMA*				
Vaizey				
Median score	6	4	7.5	7.5
Range	1–24	1–16	2–23	1–24
				
IBDQ				
Median score	191.5	215	213	207
Range	101–223	158–223	156–223	101–223
				
Small bowel SOMA				
Median score	0.3076	0.1538	0.2307	0.2307
Range	0.0769–1	0.0769–0.5384	0.0769–0.5385	0.0769–1
				
Rectal SOMA				
Median score	0.2143	0.1429	0.2143	0.2143
Range	0.0714–1	0.0714–0.7143	0.0714–0.9286	0.0714–1

IBDQ=Inflammatory Bowel Disease questionnaire; LENT=Late Effects on Normal Tissue; SOMA=Subjective, Objective, Management and Analytic; GI=gastroinstestinal.

**Table 8:** Pearson's correlation

	**Small intestine SOMA score**	**Rectum SOMA score**	**Vaizey Score**	**Total IBDQ score**
Small intestine SOMA score	1	0.643 (**)	0.452 (**)	−0.630 (**)
Rectum SOMA score	0.643 (**)	1	0.760 (**)	−0.616 (**)
Vaizey score	0.452 (**)	0.760 (**)	1	−0.599 (**)
Total IBDQ score	−0.630 (**)	−0.616 (**)	−0.599 (**)	1
Significance level (two-tailed)	<0.001	<0.001	<0.001	<0.001

IBDQ=Inflammatory Bowel Disease questionnaire; SOMA=Subjective, Objective, Management and Analytic;

** Correlation is significant at the 0.01 level (two-tailed).
